# Nicotinic Acid Improves Endurance Performance of Mice Subjected to Treadmill Exercise

**DOI:** 10.3390/metabo10040138

**Published:** 2020-04-01

**Authors:** Robert Ringseis, Denise K. Gessner, Anna M. Beer, Yvonne Albrecht, Gaiping Wen, Erika Most, Karsten Krüger, Klaus Eder

**Affiliations:** 1 Institute of Animal Nutrition and Nutrition Physiology, Justus Liebig University Giessen, Heinrich-Buff-Ring 26-32, 35392 Giessen, Germany; denise.gessner@ernaehrung.uni-giessen.de (D.K.G.); anna.m.kynast@ernaehrung.uni-giessen.de (A.M.B.); yvi.albrecht@gmx.net (Y.A.); gaiping.wen@ernaehrung.uni-giessen.de (G.W.); erika.most@ernaehrung.uni-giessen.de (E.M.); Klaus.Eder@ernaehrung.uni-giessen.de (K.E.); 2 Department of Exercise Physiology and Sports Therapy, Institute of Sports Science, Justus-Liebig-University Giessen, Kugelberg 62, 35394 Giessen, Germany; karsten.krueger@sport.uni-giessen.de

**Keywords:** adaptation, endurance, exercise, nicotinic acid, skeletal muscle, unfolded protein response

## Abstract

Recently, administration of nicotinic acid (NA) at a pharmacological dose was found to induce a similar change in the muscle´s contractile and metabolic phenotype as observed in response to endurance exercise. Thus, the hypothesis was tested that combined NA administration and endurance exercise promotes the adaptation of muscle to regular exercise and improves the endurance performance to a greater extent than exercise alone. Thus, 30 adult mice were randomly divided into three groups of 10 mice/group. The control and the exercise (EX) group received an adequate NA diet, while the EX + NA group received a high NA diet. Mice of the EX and the EX + NA group were subjected to a treadmill endurance exercise program five times/week during the experimental period of 42 days. At day 41, endurance performance was greater in the EX + NA group than in the control and the EX group (*p* < 0.05). Mice of the EX + NA group had a higher type IIA (+60%) and a lower type IIB (−55%) fiber percentage in gastrocnemius (GN) muscle than control mice (*p* < 0.05), while the type I fiber percentage in GN muscle tended to be increased (+100%) in the EX + NA group compared to the control group (*p* = 0.051). In the EX + NA group, glycogen concentration (+15%) and mRNA levels of two glycolytic (+70–80%) and two glycogenolytic enzymes (+80–120%) in GN muscle were increased compared to the control group (*p* < 0.05). In conclusion, feeding a high NA diet induces changes in skeletal muscle fiber composition and improves endurance performance of mice subjected to regular endurance exercise.

## 1. Introduction

Nicotinic acid (NA) belongs to the B-complex of vitamins which share as a common characteristic to act as precursors of specific coenzymes (NAD^+^ and NADP^+^) essentially required in intermediary metabolism in all tissues. Apart from this physiological function of NA, NA is well known to exhibit pronounced blood lipid-modulating activities, such as lowering of triacylglycerols (TAG), at high doses (in humans 2–6 g/day) [1]. This pharmacological activity of NA has long been mainly attributed to an NA receptor-mediated inhibition of lipolysis in white adipose tissue, thereby, reducing the substrate (free fatty acids (FFA)) availability for the synthesis of TAG in the liver—the main source of plasma TAG [1]. However, this mechanism can only insufficiently explain the TAG-modulating effect of NA, because plasma FFA levels during long-term NA administration typically exceed pre-treatment levels due to a marked rebound and even overshoot phenomenon, yet the TAG-lowering effect persists [2]. Against this background, recent research dealing with lipid-modulating effects of NA has also focused on skeletal muscle which does not express the NA receptor hydroxycarboxylic acid receptor 2, but owing to its great mass and extensive utilization of fatty acids as energy source affects plasma TAG concentration. In this context the observation from a recent study that feeding NA at pharmacological levels to insulin-resistant obese rats affects the contractile and metabolic phenotype of skeletal muscle is remarkable [3]. Generally, the muscle´s phenotype exhibits high plasticity in response to diverse factors. For instance, obesity decreases the percentage of oxidative type I fibers while increasing the percentage of less oxidative fibers and glycolytic fibers. In contrast, weight loss reverses these obesity-induced changes of muscle fiber composition in humans and rodents [4,5]. In the study with obese rats, it was found that an obesity-induced reduction in the percentage of type I fibers and an increase in type II fibers in muscle of obese rats is prevented by feeding a pharmacological dose of NA [3]. Type I fibers are rich in mitochondria, exhibit high activities of oxidative enzymes and are able to efficiently use fatty acids for ATP production [6]. Thus, the NA-induced change in muscle contractile phenotype of obese rats resulted in a more oxidative metabolic phenotype as evidenced by higher muscle expression of genes involved in fatty acid utilization and oxidative phosphorylation [3]. These findings suggested that an increased utilization of fatty acids by muscle might contribute to the TAG-lowering effect of NA. 

Similar changes of the muscle´s fiber distribution as observed with NA, also occur in response to regular aerobic endurance exercise, namely a muscle fiber transformation from glycolytic, fatigue-susceptible type IIB to oxidative, fatigue-resistant type IIA and type I fibers [7]. This adaptation of muscle explains to a significant extent, apart from the improvement of cardiopulmonary function, the improved endurance exercise capacity as determined by maximum oxygen uptake (VO_2_max) of individuals regularly performing endurance training. In light of the similar effects of NA administration and regular endurance exercise, we hypothesized that the combination of both promotes the adaptation of muscle to regular exercise and improves the endurance exercise performance to a greater extent than exercise alone. To test this hypothesis, mice were subjected to a six week endurance exercise program and fed with either an NA adequate diet sufficient to cover their vitamin requirement or a high NA diet providing a pharmacological NA dose which is comparable to that used for lipid-lowering in humans.

Recent studies demonstrated that activation of stress signaling pathways, such as the unfolded protein response (UPR), plays a regulatory role in the adaptation of the muscle´s phenotype to exercise [8]. The UPR is a protein quality control mechanism comprising a transcriptional program which aims at reducing global protein translation, while increasing production of selected chaperones and proteases involved in protein folding and restoration of proteostasis. The UPR is activated when unfolded and misfolded proteins accumulate in the endoplasmic reticulum (UPR^ER^), but a similar, yet independent compartment-specific UPR, the mitochondrial UPR (UPR^MT^), originates from a disturbed proteostasis within the mitochondrial matrix and intermembrane space [9]. Owing to the regulatory role of the UPR for the muscle´s adaptation to exercise, we also investigated the effect of NA treatment on the UPR^ER^ and UPR^MT^ pathways.

## 2. Results

### 2.1. Body Weight Development and Food Intake of the Mice

**Figure 1:**
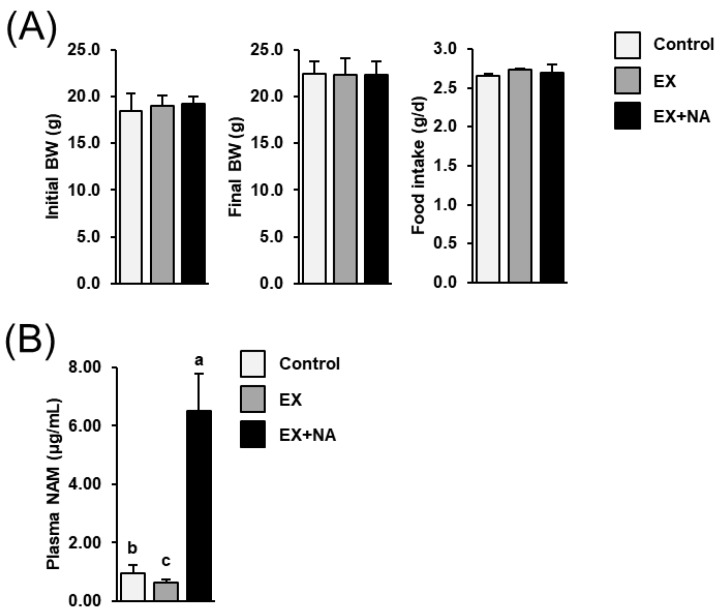
Initial and final body weights (BW) and food intake (**A**) and plasma nicotineamide (NAM) concentration (**B**) of sedentary mice fed an NA (nicotinic acid) adequate diet (Control) and mice subjected to regular endurance exercise fed either an NA adequate diet (EX) or a high NA diet (EX + NA) for 6 weeks. Bars represent means ± SD for *n*= 10 animals per group. Bars without a common lowercase letter (a, b, c) differ, *p* < 0.05.

Initial and final body weights and food intake of mice did not differ between groups (Figure 1A). Plasma concentration of the NA metabolite nicotineamide (NAM) was markedly higher in the EX + NA group compared to the control group and the EX group (Figure 1B; *p* < 0.05). In mice of the EX group, the plasma NAM concentration was lower than in mice of the control group (*p* < 0.05). The plasma concentrations of NA and the NA metabolite nicotinuric acid (NUA) in all groups could not be determined, because concentrations were lower than the limit of detection (<0.01 µg/mL).

### 2.2. Development of Endurance Exercise Capacity of the Mice

**Figure 2:**
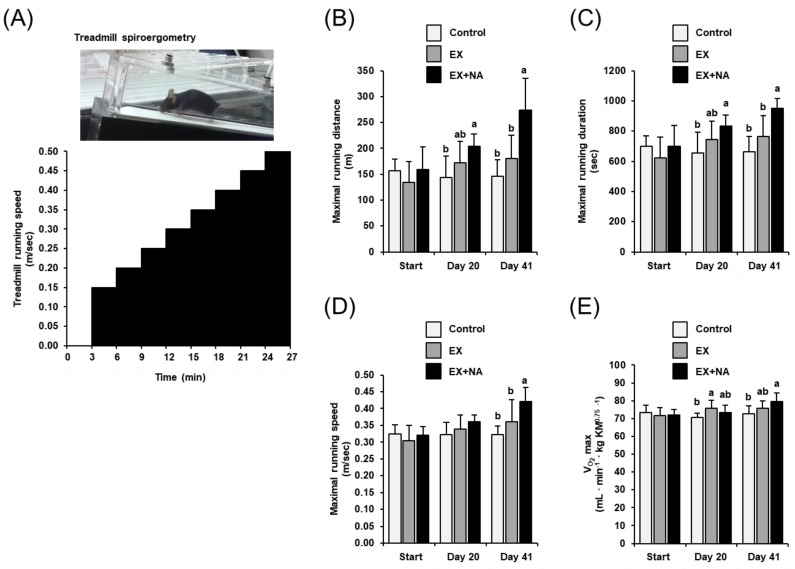
Development of endurance exercise performance in sedentary mice fed an NA (nicotinic acid) adequate diet (Control) and mice subjected to regular endurance exercise fed either an NA adequate diet (EX) or a high NA diet (EX + NA) for 6 weeks. Endurance exercise performance was determined by means of a maximal running test on a treadmill spiroergometry (**A**). Following a short equilibration phase of 3 min in the airtight treadmill at 0 m/sec, the maximal running test started at a running speed of 0.15 m/sec for 3 min followed by a step-wise increase of running speed by 0.05 m/sec every 3 min until exhaustion. As parameters of endurance exercise performance maximal running distance (**B**), maximal running duration (**C**), maximal running speed (**D**) and maximal oxygen uptake (VO_2_max) (**E**) were determined. Bars represent means ± SD for *n* = 10 animals per group. Bars without a common lowercase letter (a, b) differ, *p* < 0.05.

At the start of the experiment, parameters of endurance exercise capacity assessed by a maximal running test until exhaustion using treadmill spiroergometry as illustrated in Figure 2A did not differ between mice of all groups. At day 20 of the experiment, the maximal running distance (Figure 2B) and the maximal running duration (Figure 2C) were higher (+25–40%) in mice of the EX + NA group than in those of the control group (*p* < 0.05), whereas the maximal running speed (Figure 2D) tended to be higher (about +15%) in the EX + NA group than in the control group (*p* = 0.068). In mice of the EX group, VO_2_max was higher (+10%, *p* < 0.05, Figure 2E) and maximal running distance and maximal running duration were numerically higher than in mice of the control group at day 20 of the experiment. At day 41 of the experiment, maximal running distance, maximal running duration, maximal running speed and VO_2_max were higher (+10–80%) in the EX + NA group than in the control group (*p* < 0.05; Figure 2B–E). In the EX group, VO_2_max, maximal running distance, maximal running duration and maximal running speed at day 41 of the experiment were numerically higher than in the control group.

### 2.3. Fiber Type Composition of Gastrocnemius Muscle of the Mice

**Figure 3:**
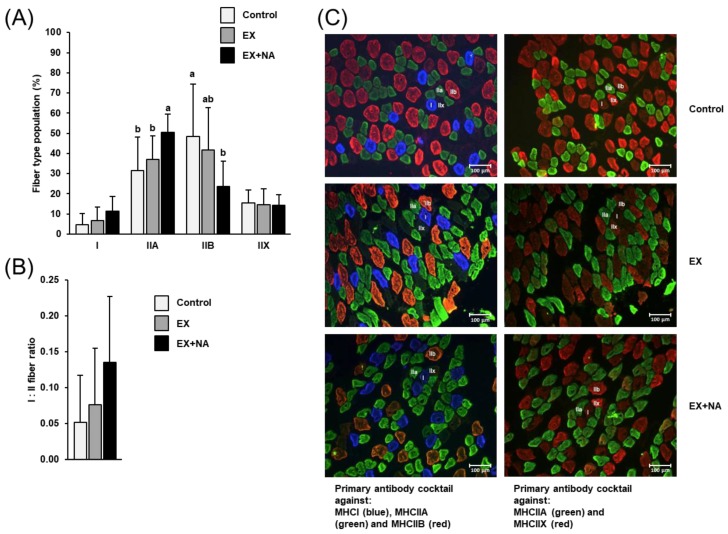
Fiber type composition (**A**) and fiber type I:type II ratio (**B**) of gastrocnemius muscle of sedentary mice fed an NA (nicotinic acid) adequate diet (Control) and mice subjected to regular endurance exercise fed either an NA adequate diet (EX) or a high NA diet (EX + NA) for 6 weeks. Bars represent means ± SD for *n* = 10 animals per group. Bars without a common lowercase letter (a, b) differ, *p* < 0.05. (**C**) Representative images were obtained from two sequential cross-sections of each muscle sample stained with two different primary antibody cocktails (left: 1, right: 2) in order to discriminate the four fiber types. In cross-section 1, blue fibers were classified as type I, green fibers as type IIA and red fibers as type IIB. In cross-section 2, green fibers were classified as type IIA and red fibers as type IIX. For fiber typing, per animal all fibers within two representative fields-of-view at a 100-fold magnification were counted and typed. Bar represents 100 µm.

In line with published literature, immunohistochemical fiber typing of gastrocnemius (GN) muscle using fluorescence-labelled antibodies revealed a strong predominance of type II fibers (90–95%; Figure 3A,C). Despite the low percentage of type I fibers in GN muscle (5–10%), the percentage of type I fibers tended to be increased by about 100% in mice of EX + NA group compared to the control group (*p* = 0.051; Figure 3A,C). In addition, the ratio of type I to II fibers tended to be about 130% higher in mice of the EX + NA group than in mice of the control group (*p* = 0.051; Figure 3B). Moreover, mice of the EX + NA group had a higher (+60%) percentage of type IIA fibers and lower (−55%) percentage of type IIB fibers in GN muscle than mice of the control group (*p* < 0.05, Figure 3A,C), whereas no difference was observed between the three groups in the percentage of type IIX fibers. In the EX group, the percentage of type IIB fibers was numerically lower (−15%) than in the control group, but this effect was not significant. The other fiber type percentages did not differ between the EX group and the control group.

### 2.4. Expression of Genes involved in Mitochondrial Biogenesis and Genes Encoding Mitochondrial Enzymes in GN Muscle of the Mice

**Figure 4:**
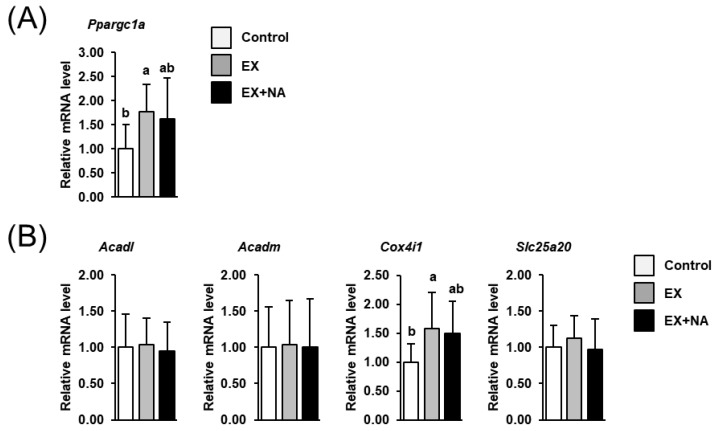
Relative mRNA levels of genes involved in mitochondrial biogenesis- (**A**), peroxisome proliferator-activated receptor gamma coactivator 1 alpha (*Ppargc1a*)) and genes encoding mitochondrial enzymes (**B**), acyl-CoA dehydrogenase long chain (*Acadl*); acyl-CoA dehydrogenase medium chain (*Acadm*); cytochrome c oxidase subunit 4I1 (*Cox4i1*); solute carrier family 25 member 20 (*Slc25a20*)) in gastrocnemius muscle of sedentary mice fed an NA adequate diet (Control) and mice subjected to regular endurance exercise fed either an NA adequate diet (EX) or a high NA diet (EX + NA) for 6 weeks. Bars represent means ± SD for *n* = 10 animals per group. Relative mRNA levels are expressed as fold of Control (= 1.0). Bars without a common lowercase letter (a, b) differ, *p* < 0.05.

The mRNA level of the key regulator of mitochondrial biogenesis peroxisome proliferator-activated receptor gamma coactivator 1 alpha (*Ppargc1a*) was higher (+78%) in mice of the EX group than in those of the control group (*p* < 0.05, Figure 4A). In the EX + NA group, the mRNA level of *Ppargc1a* tended to be higher (+62%) than in the control group (*p* = 0.090). The mRNA levels of genes involved in mitochondrial fatty acid import [solute carrier family 25 member 20 (Slc25A20)] and mitochondrial fatty acid β-oxidation [acyl-CoA dehydrogenase long chain (Acadl), acyl-CoA dehydrogenase medium chain (Acadm)] in GN muscle were not different across the three groups (Figure 4B). However, the mRNA level of the respiratory chain complex cytochrome c oxidase subunit 4I1 (Cox4i1) was significantly higher (+58%, *p* < 0.05) and tended to be higher (+49%, *p* = 0.067) in the EX group and the EX + NA group, respectively, than in the control group (Figure 4B).

### 2.5. Plasma Concentrations of Metabolites Related to Fatty Acid Metabolism in the Mice

**Figure 5:**
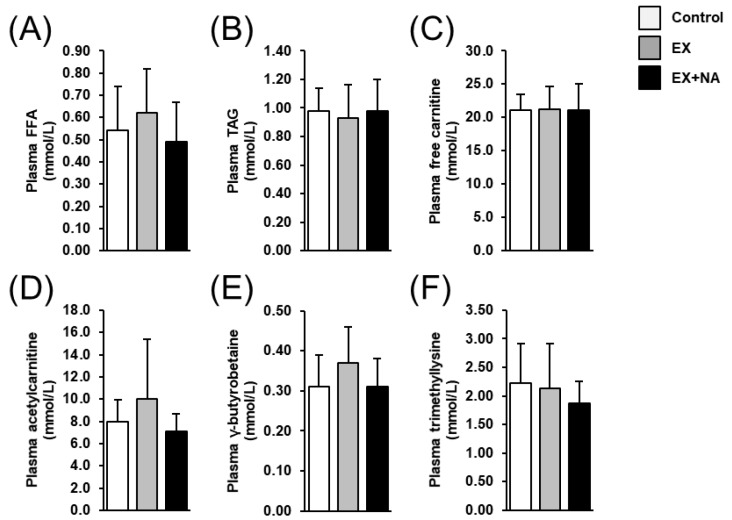
Plasma concentrations of metabolites related to fatty acid metabolism (**A**), free fatty acids (FFA); (**B**), triacylglycerols (TAG; (**C**), free carnitine; (**D**), acetylcarnitine; (**E**), γ-butyrobetaine; (**F**), trimethyllysine) in sedentary mice fed an NA (nicotinic acid) adequate diet (Control) and mice subjected to regular endurance exercise fed either an NA adequate diet (EX) or a high NA diet (EX + NA) for 6 weeks. Bars represent means ± SD for *n* = 10 animals per group.

Plasma concentrations of FFA, TAG, free carnitine, acetylcarnitine and carnitine precursors (γ-butyrobetaine, trimethyllysine) did not differ across the three groups (Figure 5).

### 2.6. Expression of Genes Encoding Enzymes of Carbohydrate Metabolism and Concentration of Glycogen in GN Muscle of the Mice 

**Figure 6:**
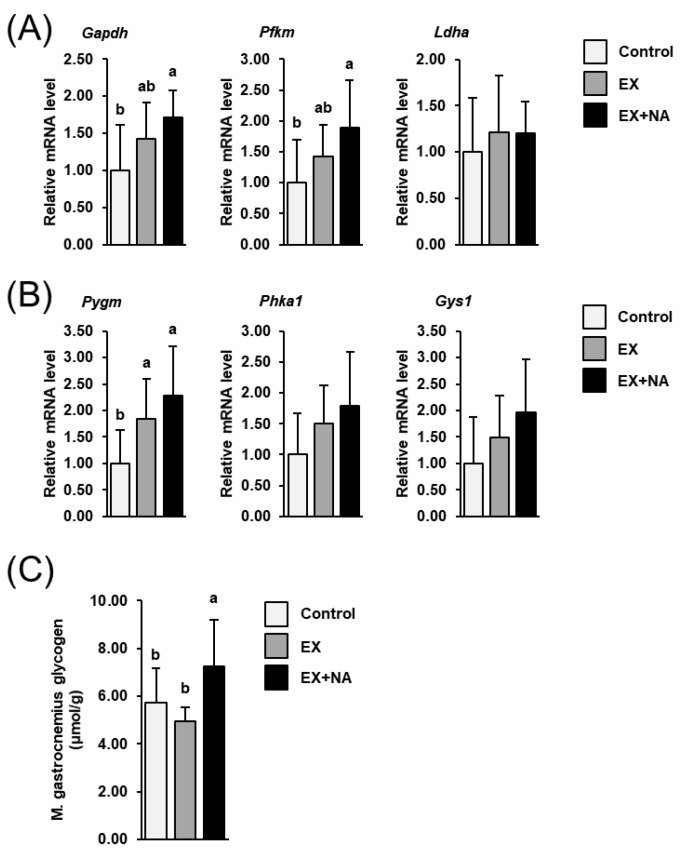
Relative mRNA levels of glycolytic genes (**A**), glyceraldehyde-3-phosphate dehydrogenase (*Gapdh*); phosphofructokinase, muscle (*Pfkm*); lactate dehydrogenase A (*Ldha*)) and genes involved in glycogenolysis and glycogen synthesis (**B**), glycogen phosphorylase, muscle associated (*Pygm*); phosphorylase kinase regulatory subunit alpha 1 (*Phka1*); glycogen synthase 1 (*Gys1*)) and glycogen concentration (**C**) in gastrocnemius muscle of sedentary mice fed a nicotinic acid (NA) adequate diet (Control) and mice subjected to regular endurance exercise fed either an NA adequate diet (EX) or a high NA diet (EX + NA) for 6 weeks. Bars represent means ± SD for *n* = 10 animals per group. Relative mRNA levels are expressed as fold of Control (= 1.0). Bars without a common lowercase letter (a, b) differ, *p* < 0.05.

The mRNA levels of two genes encoding rate-limiting glycolytic enzymes (glyceraldehyde-3-phosphate dehydrogenase (Gapdh), phosphofructokinase, muscle (Pfkm)) were increased by about 70–80% in GN muscle of mice of the EX + NA group compared to mice of the control group (*p* < 0.05, Figure 6A). In mice of the EX group, the mRNA levels of Gapdh and Pfkm in GN muscle were numerically higher (about +40%) than in mice of the control group, but this effect was not significant. The mRNA level of the glycolytic enzyme lactate dehydrogenase A (Ldha) in GN muscle did not differ across the three groups of mice. The mRNA levels of the rate-limiting glycogenolytic enzyme glycogen phosphorylase, muscle associated (Pygm) and of another glycogenolytic enzyme phosphorylase kinase regulatory subunit alpha 1 (Phka1) in GN muscle were increased (about +120%, *p* < 0.05, Figure 4B) and tended to be increased (about +80%, *p* = 0.084, Figure 6B), respectively, in the EX + NA group compared to the control group. In the EX group, the mRNA level of Pygm in GN muscle was also higher (about +80%) than in the control group (*p* < 0.05, Figure 4B). The mRNA level of the rate-limiting enzyme of glycogen synthesis glycogen synthase 1 (Gys1) in GN muscle tended to be increased (about +90%) in the EX + NA group compared to the control group (*p* = 0.083, Figure 4B).

The concentration of glycogen in GN muscle was higher (about +15%) in mice of the EX + NA group than in mice of the control group and the EX group (*p* < 0.05, Figure 6C). The latter two groups did not differ with regard to the glycogen concentration in GN muscle.

### 2.7. Expression of Genes Involved in the Cellular Stress Response in GN Muscle of the Mice

**Figure 7:**
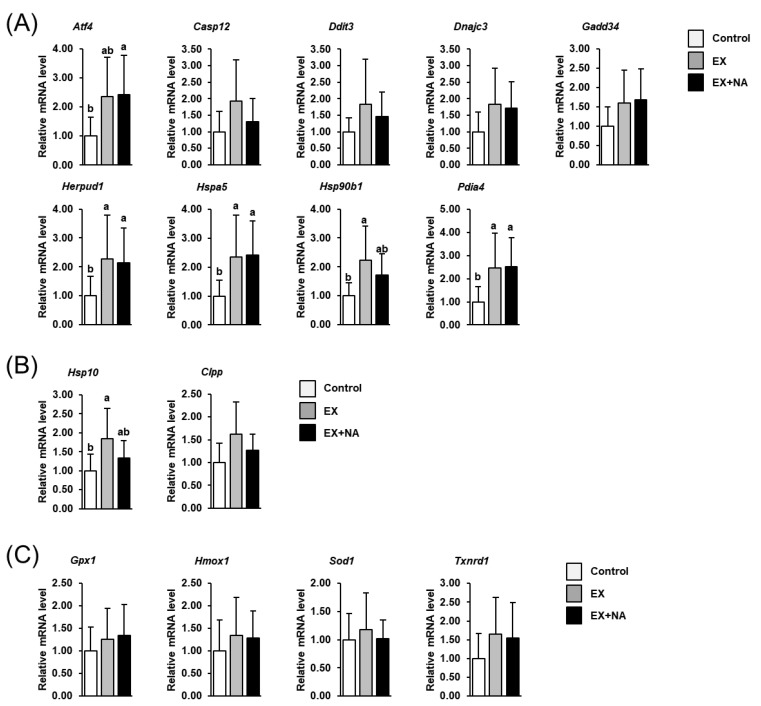
Relative mRNA levels of UPR^ER^ target genes (**A**), activating transcription factor 4 (*Atf4*); caspase 12 (*Casp12*); DNA damage inducible transcript 3 (*Ddit3*); DnaJ heat shock protein family (Hsp40) member C3 (*Dnajc3*); growth arrest and DNA-damage-inducible 34 (*Gadd34*); homocysteine inducible ER protein with ubiquitin like domain 1 (*Herpud1*); heat shock protein family A (Hsp70) member 5 (*Hspa5*); heat shock protein 90 beta family member 1 (*Hsp90b1*); protein disulfide isomerase family A member 4 (*Pdia4*)), UPR^MT^ target genes (**B**), heat shock protein family E (Hsp10) member 1 (*Hsp10*); caseinolytic mitochondrial matrix peptidase proteolytic subunit (*Clpp*)) and cytoprotective genes involved in the antioxidant response (**C**), glutathione peroxidase 1 (*Gpx1*); heme oxygenase 1 (*Hmox1*); superoxide dismutase 1 (*Sod1*); thioredoxin reductase 1 (*Txnrd1*)) in gastrocnemius muscle of sedentary mice fed an NA adequate diet (Control) and mice subjected to regular endurance exercise fed either an NA adequate diet (EX) or a high NA diet (EX + NA) for 6 weeks. Bars represent means ± SD for *n* = 10 animals per group. Relative mRNA levels are expressed as fold of Control (=1.0). Bars without a common lowercase letter (a, b) differ, *p* < 0.05.

The mRNA levels of several UPR^ER^ target genes (activating transcription factor 4 (Atf4), homocysteine inducible ER protein with ubiquitin like domain 1 (Herpud1), heat shock protein family A (Hsp70) member 5 (Hspa5), heat shock protein 90 beta family member 1 (Hsp90b1), protein disulfide isomerase family A member 4 (Pdia4)) in GN muscle were increased by about 80%–120% in mice of the EX group and the EX + NA group compared to the CON group (*p* < 0.05, Figure 7A), but the mRNA levels of these genes did not differ between the EX group and the EX + NA group. The mRNA levels of the other UPR^ER^ target genes (caspase 12 (Casp12), DNA damage inducible transcript 3 (Ddit3), DnaJ heat shock protein family (Hsp40) member C3 (Dnajc3), growth arrest and DNA-damage-inducible 34 (Gadd34)) did not differ between groups. Amongst the UPR^MT^ target genes, only the mRNA level of heat shock protein family E (Hsp10) member 1 (Hsp10) was higher (about +80%) in the EX group than in the CON group (*p* < 0.05, Figure 7B), whereas the mRNA level of Hsp10 did not differ between the EX + NA group and the CON group. The mRNA level of the other UPR^MT^ target gene (caseinolytic mitochondrial matrix peptidase proteolytic subunit (Clpp)) was not different across the groups. The mRNA levels of genes involved in the antioxidant defense system (glutathione peroxidase 1 (Gpx1), heme oxygenase 1 (Hmox1), superoxide dismutase 1 (Sod1), thioredoxin reductase 1 (Txnrd1)) in GN muscle did not differ across the groups (Figure 7C).

## 3. Discussion

The main finding of the present study is that administration of a high NA diet to mice subjected to a six week endurance exercise program improves endurance exercise performance. The NA dose ingested from the high NA diet caused a 7- to 10-fold elevation in the plasma NAM concentration in mice of the EX + NA group compared to mice of the other groups. The NA dose administered to the mice (approximately 95 mg/kg body weight) is comparable to that used in humans, in which NA doses of 2–6 g/day equivalent to 30–90 mg/kg body weight for an individual weighing 70 kg are applied to induce serum lipid-lowering effects. In agreement with other studies, NA and NUA could not be detected even in plasma of mice fed the high NA diet [3,10]. This is attributed to the fact that dietary NA is rapidly converted to NAD^+^ in the intestine and liver, and NAD^+^ is then transformed into NAM via the “amidation pathway” and subsequently released into the blood stream [11]. 

A further key finding of the present study is that fiber type composition of GN muscle—a phasic muscle strongly involved in the movement of the hindleg during treadmill running—in mice of the EX + NA group exhibited an increased percentage of oxidative fibers (fast-twitch type IIA), which mainly utilize oxidative metabolism for ATP production and thus are fatigue-resistant, compared to the control group and the EX group. In addition, mice of EX + NA group had a decreased percentage of fast-twitch type IIB fibers, which rely mainly on glycolytic metabolism for ATP production and are susceptible to exhaustion, and a trend towards a higher type I to type II-ratio compared to control group. This kind of transformation from type IIB to type IIA and type I fibers observed in the EX + NA group typically occurs as an adaptation to regular exercise training [12], and is provoked mainly by a transformation from type IIB to IIA and to a fewer extent to type I [13]. In line with this, treadmill running exercise was reported to increase the fiber type IIA percentage, to decrease the fiber type IIB percentage and to numerically increase the fiber type I percentage in rat plantaris muscle [13], which has a similar fiber type composition as GN muscle. Similar adaptations of fiber type composition of extensor digitorum longus muscle, which also predominantly contains type II fibers, to running exercise were reported from Xu et al. [14]. In contrast, no adaptations of muscle fiber composition to regular exercise training have been found in muscles containing predominantly type I fibers, such as soleus muscle [13,14]. In the present study, mice of the EX group exhibited a similar transformation of muscle fiber types as mice of the EX + NA group, but the transformation was less strong and thus the change of fiber type composition was not statistically significant. Given that the adaptation of muscle fiber type composition to exercise training is a long-term effect, it is possible that the 6-week-training protocol was not long enough to induce a significant fiber transformation as seen after 12 weeks of training [14]. However, the observation that fiber type transformation was more pronounced in mice of the EX + NA group than in mice of the EX group may be explained by an accelerated adaptation of muscle to regular exercise training by NA. Possibly, the recently observed stimulatory effect of NA on molecular regulators of fiber type transformation such as PGC-1α [3,15] may be causative for an accelerated fiber type transformation in mice of the EX + NA group. An up-regulation of PGC-1α in muscle and a type II to type I fiber transformation in response to administration of NA was also observed in pigs [16], indicating that activation of PGC-1α is a consistent effect of NA. In the present study, the mRNA level of *Ppargc1a* (encoding PGC-1α) was at least numerically elevated in GN muscle of the EX + NA group compared to the control group, but this elevation was not statistically significant. However, since the mRNA level of *Ppargc1a* in GN muscle of the EX group was similarly elevated as in the EX + NA group, it is unlikely that induction of *Ppargc1a* is causative for the accelerated fiber type transformation in the EX + NA group. Nevertheless, our observations with regard to *Ppargc1a* mRNA should not be overstated, because the mRNA level of this transcription factor is not an indicator of its function.

In order to elucidate if the fiber type transformation of GN muscle was accompanied by changes of the metabolic phenotype, we analyzed the expression of key genes involved in important energy generating pathways. It is important to note that the effects observed at the mRNA level largely reflect the adaptation of muscle to the six-week-intervention period and not the acute response to the strenuous running test, because the mice were euthanized two days after the last exercise bout. While the expression of genes involved in fatty acid utilization in GN muscle did not differ across the three groups of mice, it was obvious that genes involved in glycolysis (*Gapdh*, *Pfkm*) and glycogenolysis (*Pygm*) were increased in GN muscle of the EX + NA group. In line with the unaltered expression of genes involved in fatty acid oxidation in GN muscle, plasma concentrations of metabolites related to fatty acid metabolism were not different across the groups. In contrast, the concentration of glycogen in GN muscle was elevated in the EX + NA group compared to the control group, but not in the EX group. An elevated concentration of glycogen and an increased mRNA level of *Pygm* in GN muscle at rest was also reported in rats which had access to voluntary wheel running for eight weeks [17], which is indicative of a well-known training effect (“glycogen loading”) from running activity. Even in an “oxidative” muscle containing mainly slow-twitch type I fibers such as soleus muscle, voluntary wheel running for 12 weeks was found to increase intramuscular glycogen concentration [14]. Because intramuscular glycogen concentration allows the muscle to generate ATP from glucose under anaerobic conditions, it is likely that the increased glycogen levels in GN muscle of the EX + NA group contributed to the higher resistance to exhaustion during the progressive treadmill running test, because at the higher speed steps the mice had to run in the anaerobic range. Apart from its effect on anaerobic potential, muscle glycogen has been generally recognized to play a decisive role in regulating the capacity to sustain exercise at a given work load [18]. In line with this, glycogen depletion in muscle has been identified as a major cause of fatigue during endurance exercise at moderate to high intensities [19], and graded reductions in pre-exercise muscle glycogen concentration were recently reported to impair exercise capacity in humans [20]. Apart from the increase of muscle glycogen, the induction of both key glycogenolytic and glycolytic genes in GN muscle of the EX + NA group compared to the control group is likely an indication of an improved ability to mobilize and utilize intramuscular glucose which presumably also contributed to the higher performance of the mice during the strenuous progressive running test. In mice of the EX group, the mRNA levels of *Gapdh* and *Pfkm* tended to be increased and that of *Pygm* in GN muscle was significantly increased compared to mice of the control group—effects which are also indicative of an adaptation to regular endurance exercise in this group. Regarding the lack of increase of intramuscular glycogen concentration in this group, it is possible that the six-week-training period alone was too short to cause an increase of intramuscular glycogen stores, whereas the additional feeding of a high NA diet accelerated the adaptation to regular endurance exercise. Interestingly, despite increased secretion of lipolytic hormones, NA administration causes potent anti-lipolytic effects in white adipose tissue [2], which explains that acute administration of a pharmacological NA dose in humans and rodents causes a rapid decrease in plasma FFA levels. This rapid decrease of plasma FFA levels in response to acute NA administration, however, is followed by a marked rebound and even an overshoot of plasma FFA levels above pre-treatment levels [2]. Accordingly, chronic NA administration results in elevated basal and fasting FFA levels in plasma [21]. It is thus possible that supplementing the mice with the high NA dose increased intramuscular availability of FFA due to an elevated flow of FFA into the exercising muscle. The observation that plasma FFA levels were at least numerically lower in the EX + NA group than in the EX group might be indicative of an increased utilization of the FFA by the exercising muscle in these mice. Thus, an increased availability of both, glucose from glycogen and FFA in exercising muscle might have contributed to the higher resistance to exhaustion during the progressive treadmill running test.

Recent studies demonstrated that the UPR, a cellular stress signaling pathway which can emanate from the endoplasmic reticulum (UPR^ER^) and from the mitochondrion (UPR^MT^), is activated in muscle in response to the exercise stimulus and plays an essential regulatory role in the muscle´s adaptation to regularly exercise [8]. In fact, genetic blockade of UPR activation was shown to impair recovery from exhaustive treadmill running and to cause exercise intolerance after repetitive treadmill running bouts [8]. Activation of the UPR in response to exercise has been reported to be related to a stimulation of mitochondrial biogenesis mediated by different exercise signals like intracellular Ca^2+^ concentration and levels of reactive oxygen species (ROS) or cAMP [22,23]. In the present study, the expression of several UPR^ER^ and UPR^MT^ target genes was found to be increased in GN muscle of mice in the EX group and the EX + NA group compared to the CON group, but the expression of these genes did not differ between the EX group and the EX + NA group. Thus, these data suggest that the metabolic adaptations and the improved performance of mice of the EX + NA group compared to EX group are not mediated by an alleviation of the UPR pathway in the trained skeletal muscle. However, NA administration is well known to cause several humoral changes, such as increases in the plasma levels of lipolytic hormones like adrenalin and corticosterone and modulates circulating levels of growth hormone and adiponectin [1,15,24,25], all of which may affect skeletal muscle´s supply with energy substrates and thus exercise performance. In addition, it is also possible that NA administration has improved mitochondria function through an increased provision of NAD^+^, which is rapidly formed from NA in tissues following intestinal absorption of NA. As already mentioned above, dietary NA is rapidly converted to NAD^+^ in the intestine and liver before it is transformed into NAM. Thus, it is very likely that tissue levels of NAD^+^ have been increased, at least transiently, in the mice fed the high NA diet. Unfortunately, we were not capable of determining intramuscular levels of NAD^+^, but the critical role of this metabolite for mitochondria function is beyond question. Besides acting as an essential electron carrier in numerous energy-generating pathways including glycolysis, β-oxidation and tricarboxylic acid cycle, NAD^+^ has been shown to maintain mitochondria function through affecting multiple NAD^+^-dependent protein deacetylases, poly(ADP-ribose) polymerases and transcription factors [26].

One limitation of this study is the lack of a group of mice, which received the high NA diet but were not subjected to exercise training. This would have enabled us to study the main effect of NA administration and thus to evaluate if some of the alterations observed in the EX + NA group are primarily caused by the administration of NA. As mentioned above, pharmacological levels of NA as administered via the high NA diet cause profound effects on circulating levels of various hormones, which might have interfered with the production of intramuscular levels of exercise signals, thereby, affecting the adaptation of muscle to regular exercise in the EX + NA group. Future studies have to clarify the role of NA in this scenario. In addition, a further limitation of the study is that the three treatment groups did not include a subgroup of mice, which were sacrificed immediately after the final acute running test. This would have allowed us to additionally investigate the metabolic response to the acute strenuous exercise bout and its potential modulation by NA administration. This could have provided a deeper insight into the metabolic adaptations underlying the performance-improving effect of NA treatment.

In conclusion, the present findings suggest that administration of NA at pharmacological levels increases endurance exercise performance of trained mice through improving the adaptation of muscle to regular exercise. Whether the exercise performance-improving effect of NA might also involve other tissues than skeletal muscle like the heart, which significantly affects endurance exercise capacity through regulating the stroke volume, and other factors determining endurance exercise capacity (e.g., red blood cell volume, hematocrit) cannot be answered from this study. As VO_2_max, the gold standard parameter of cardiorespiratory fitness, is strongly dependent on stroke volume and red blood cell volume [27], we cannot rule out that improvements of heart function and/or an expansion of red blood cell volume might have also contributed to the beneficial effect of NA administration on endurance exercise performance. This has to be clarified in future studies.

## 4. Materials and Methods 

### 4.1. Animals

The animal experiment was approved by the local Animal Care and Use Committee (Regierungspräsidium Giessen; permission no: JLU 16/2013). All experimental procedures described followed established guidelines for the care and handling of laboratory animals. 30 female, 10-wk-old C57BL/6N mice were purchased from Charles River (Sulzfeld, Germany) and housed in groups of five animals each under controlled conditions (12 h light:12 h dark, 22 ± 1 °C ambient temperature, 50–60% relative humidity).

### 4.2. Diets

At the day of the experimental start, the mice were randomly divided into three groups (Control, EX, EX + NA) of 10 mice each. The mice were fed two different semi-purified diets, whose nutrient concentrations were sufficient to meet requirements of growing mice according to National Research Council (NRC) [28]. The first diet (“adequate NA diet”) containing 30 mg supplemented NA/kg diet, which was sufficient to cover the NA requirement according to NRC [28], was fed to the control group and the EX group, whereas the second diet (“high NA diet”) containing 780 mg supplemented NA/kg diet (Lonza, Basel, Switzerland) was fed to the EX + NA group (Table 1). All diet components were obtained from different sources (Supplementary Table S1). The diets were prepared by mixing the components and subsequent pelleting with a standard pelleting device (Kahl Laborpressanlage Typ 14–175; Reinbek, Germany). The mice had free access to the experimental diets which were fed for 42 days. Water was constantly available ad libitum from nipple drinkers.

### 4.3. Determination of Endurance Exercise Performance

Owing to their nocturnal lifestyle, exercise capacity of mice of all groups was determined in the dark phase, the activity period of mice, two days before the start of the experiment and at days 20 and 41 of the experiment using treadmill spiroergometry. The treadmill spiroergometry consisted of a computer-controlled airtight single-lane mouse treadmill (without shock grid), a multichamber air supplier (LE400) and a gas analyzer measuring O_2_ and CO_2_ concentrations in the air (LE405; all from Harvard Apparatus, Holliston, MA, USA). As parameters of endurance exercise performance, VO_2_max, maximal running speed, maximal running distance and maximal running duration were determined by means of a maximal running test on the treadmill (set at 25% incline). In detail, following a short equilibration phase of 3 min in the airtight treadmill at 0 m/sec, the maximal running test started at a running speed of 0.15 m/sec for 3 min followed by a step-wise increase of running speed by 0.05 m/sec every 3 min until exhaustion. Exhaustion was defined as the point at which mice stopped running and fell back to the lower end of the treadmill at least two times. Maximal running speed, maximal running distance and maximal running duration were recorded at exhaustion.

### 4.4. Endurance Exercise Program

Mice of the EX group and the EX + NA group were subjected to a controlled endurance exercise program during the experimental phase which was performed during the dark phase on a five-lane mouse treadmill (without shock grid) from Harvard Apparatus (Holliston, MA, USA) set at an incline of 12%. In order to avoid physiological stress, mice were accustomed to the treadmill during the week before the start of experiment by running on the treadmill for ten min at a treadmill speed of 0.05 m/sec on five consecutive days. The endurance exercise program was carried out five times per week (Monday to Friday) during the experimental period of 42 days, with each exercise session lasting 35 min at a running speed corresponding to 80% of VO_2_max determined two days before the start of the experiment. To adjust the running speed to the improvement of VO_2_max during the experiment, the VO_2_max and other endurance exercise biomarkers of all mice was determined again at day 20 of the experiment.

### 4.5. Sample Collection

In order to avoid acute effects of the exhausting endurance capacity test, the mice were decapitated under CO_2_ anesthesia in the non-fasted state at day 43 of the experiment (two days after the last endurance exercise capacity test). Blood was collected into heparin-coated polyethylene tubes (Sarstedt, Nümbrecht, Germany) and plasma obtained by centrifugation at 1100× *g* for 10 min at 4 °C. The gastrocnemius (GN) muscle was prepared and excised from the right hind leg and washed in ice-cold NaCl solution (0.9%). Plasma samples and muscle aliquots (for qPCR analysis) were snap-frozen in liquid nitrogen and stored at −80 °C pending analysis.

### 4.6. Immunohistochemical Muscle Fiber Typing Using Fluorescence-Labelled Antibodies

The GN muscle of all mice was crosscut in the middle of the muscle belly into two halves and one half was embedded in Tissue Tek optimal cutting temperature (O.C.T.) compound (Sakura Finetek Germany GmbH, Staufen, Germany), frozen in liquid nitrogen and cut into serial 10 µm thick cross-sections with a microtome (Microm HM 500, Microm International, Walldorf, Germany) maintained at −20 °C. Fiber typing was carried out based on myosin heavy chain (MHC) distribution using multicolor immunofluorescence analysis as described recently by our group [29]. Two sequential cross-sections of each muscle sample were stained with two different primary antibody cocktails (1 and 2) in order to discriminate the four fiber types. Both air dried and methanol fixed cross-sections were incubated with the cocktail of primary antibodies (all purchased from Developmental Studies Hybridoma Bank, University of Iowa, USA) in 1× PBS containing 0.5% Triton X-100 and 10% goat serum at 4 °C overnight. The primary antibody cocktail 1 contained primary antibodies against MHC I (BA-F8, 1:50 dilution), MHC IIA (SC-71, 1:600 dilution) and MHC IIB (BF-F3, 1:100 dilution) and cocktail 2 contained primary antibodies one against MHC IIA (SC-71, 1:600 dilution) and MHC IIX (6H1, 1:50 dilution). Following a washing step with 1x PBS, the cross-sections were incubated with two different cocktails of fluorescent-conjugated secondary antibodies purchased from Invitrogen (Darmstadt, Germany) in 1xPBS containing 0.5% Triton X-100 and 10% goat serum at RT for two hours. The secondary antibody cocktail 1 for cross-section 1 contained Alexa Fluor 350 IgG2b (blue, 1:500 dilution) for BA-F8, Alexa Fluor 488 IgG1 (green, 1:500 dilution) for SC-71 and Alexa Fluor 555 IgM (red) for BF-F3. Secondary antibody cocktail 2 for cross-section 2 contained Alexa Fluor 488 IgG1 (green, 1:500 dilution) for SC-71 and Alexa Fluor 555 IgM (red) for 6H1. Cross-sections were washed again with 1x PBS, and then mounted in Aqua Polymount (Polyscience, Niles, Illinois) and covered with coverslips. Visualization of slides was carried out with a DM 5500 B fluorescence microscope from Leica (Wetzlar, Germany) equipped with appropriate red, green and blue filters [29], a Leica DFC340FX camera and the Leica LAS AF microscope software. Individual images were taken from the slides with each filter and a composite image was assembled automatically from the images taken with the three filters. For fiber typing, per animal all fibers within two representative fields-of-view at a 100-fold magnification were counted and typed. In cross-section 1 blue fibers were classified as type I, green fibers as type IIA and red fibers as type IIB. In cross-section 2 green fibers were classified as type IIA and red fibers as type IIX. For each animal a total of 133 ± 39 (mean ± SD) fibers were counted/typed.

### 4.7. RNA Isolation and qPCR Analysis

Total RNA isolation from 25 mg aliquots of GN muscle and cDNA synthesis was carried out as described recently in detail [30]. The mRNA levels of selected genes were measured with a Rotor-Gene Q system (Qiagen, Hilden, Germany) using KAPA™ SYBR^®^ FAST qPCR Mastermix (Peqlab, Erlangen, Germany) and gene-specific primer pairs (Eurofins MWG Operon, Ebersberg, Germany) were designed as described recently [30]. Characteristics of primers used for qPCR analysis are shown in Supplementary Table S2. Normalization using geometric averaging of multiple internal control genes and calculation of gene expression data was carried out as described [30]. The most stable reference genes were beta-2-microglobulin (B2m) and peptidylprolyl isomerase (Ppia). The mean normalized 2^−ΔCt^ ratios of the control group was set to 1.0 and means and SD of normalized 2^−ΔCt^ ratios of the other groups (EX, EX + NA) were scaled proportionally.

### 4.8. Determination of Biochemical Parameters

Plasma concentrations of NA, NAM and NUA in plasma were determined by LC-MS/MS as published recently in detail [10]. The concentrations of free carnitine, acetylcarnitine and the carnitine precursors trimethyllysine and γ-butyrobetaine in plasma were measured by tandem mass spectrometry as published recently [31]. The plasma concentrations of TAG and FFA were measured using enzymatic reagent kits from Analyticon Biotechnologies (Fluitest TG; Lichtenfels, Germany) and Wako Chemicals (NEFA-HR(2); Neuss, Germany), respectively, according to the manufacturer´s protocol. Determination of glycogen in GN muscle was carried out as described by Dawson et al. [32]. In brief, a 20–30 mg aliquot of GN muscle was incubated in 2 N HCl and heated for 2 h at 100 °C to hydrolyze the glycogen to glucosyl units. After cooling to RT, the solution was neutralized by the addition of 4 N NaOH and centrifuged at 1100× *g* for 10 min at RT. Subsequently, glucose concentration was determined in the supernatant using an enzymatic kit from Analyticon Biotechnologies (Fluitest GLU HK).

### 4.9. Statistical Analysis

All data are shown as means with their standard deviations (SD). All data were checked for normal distribution by the Anderson-Darling test and analysed by one-way ANOVA using the Minitab Statistical Software (Rel. 13.1, State College, PA, USA). Data with unequal variances were log transformed before statistical analysis. Means of the three groups were compared by Fisher’s multiple range tests. Different means are indicated by different lowercase letters. Means were considered significantly different for *p* < 0.05. A tendency towards an effect was mentioned in the text in the case that the *p*-value was < 0.1.

## Figures and Tables

**Table 1:** Composition of the experimental diets.

	Adequate NA Diet (g/kg) (30 mg niacin/kg diet)	High NA Diet (g/kg) (780 mg niacin/kg diet)
Maize starch	532	532
Casein	200	200
Saccharose	100	100
Soybean oil	70	70
Cellulose	50	49.25
Mineral mix ^1^	35	35
Vitamin mix ^2^	10	10
L-Cystein	3	3
Supplemental NA	-	0.75

^1^ The mineral mix provided the following per kg diet: calcium, 4.3 g; potassium, 3.06 g; phosphorus, 1.34 g; sodium, 0.87 g; magnesium, 0.43 g; chloride, 0.5 g; iron, 35 mg; zinc, 25 mg; manganese, 10 mg; copper, 6 mg; chromium, 1 mg; fluor, 0.9 mg; boron, 0.50 mg; nickel, 0.50 mg; iodine, 0.15 mg; molybdenum, 0.15 mg; selenium, 0.15 mg; lithium, 0.10 mg. ^2^ The vitamin mix provided the following per kg diet: vitamin A, 4000 IE; vitamin D_3_, 1000 IE; vitamin K_3_, 0.75 mg, vitamin E, 75 IE; vitamin B_1_, 5 mg; vitamin B_2_; 6 mg; vitamin B_6_, 6 mg; vitamin B_12_, 0.035 mg; biotin, 0.2 mg; folic acid, 2.0 mg; NA, 30 mg; choline, 1250 mg; pantothenic acid, 15 mg.

## Supplementary Materials

The following are available online at https://www.mdpi.com/2218-1989/10/4/138/s1, Table S1: Sources of diet components; Table S2: Characteristics of gene specific primers used for qPCR.
