# Supplementary material for: Nicotinic Acid Improves Endurance Performance of Mice Subjected to Treadmill Exercise
Source: Metabolites. 2020 Apr 1;10(4):138. doi: 10.3390/metabo10040138 (PMC7240961; doi:10.3390/metabo10040138)
Supplement: Supplementary file 1 [file metabolites-10-00138-s001.pdf]

Supplementary materials

Table S1 Sources of diet components

Component (product name)	Source (Company, City, Country)
Maize starch (Maisita – Native Maisstärke)	Agrana Beteiligungs-AG, Gmünd, Austria
Casein (Nährkasein, Säurekasein 110 Mesh)	Meggle, Wasserburg, Germany
Saccharose (Puderzucker)	Suedzucker, Mannheim, Germany
Soybean oil (Heidenreich – Soja 3-Plus)	Karl Heidenreich GmbH, Mannheim, Germany
Cellulose (Arbocel – Rohfaserkonzentrat, Typ R)	Rettenmaier & Söhne GmbH + Co KG, Rosenberg, Germany
Mineral mix[1]	Self-made
Vitamin mix[2]	Self-made
L-Cystein	Sigma Aldrich, St. Louis, USA
Supplemental NA	Lonza, Basel, Switzerland

[1]Components mineral mix

$MgSO_4$ x $7H_2O$	Roth, Karlsruhe, Germany
$CaCO_3$	Bergmann, Kasendorf, Germany
NaCl	Roth, Karlsruhe, Germany
$FeSO_4$ x $7H_2O$	Merck, Darmstadt, Germany
$ZnSO_4$ x $7H_2O$	Grüssing, Vilsum, Germany
$MnSO_4$ x H_2O	Roth, Karlsruhe, Germany
$CuSO_4$ x $5H_2O$	Roth, Karlsruhe, Germany
KI	Sigma Aldrich, St. Louis, USA
NaFl	Merck, Darmstadt, Germany
$CrK(SO_4)_2$ x $12H_2O$	Merck, Darmstadt, Germany
LiCl	Sigma Aldrich, St. Louis, USA
Na_2MoO_4 x $2H_2O$	Acros organics, New Jersey, USA
Na_2SeO_3 x $5H_2O$	Merck, Darmstadt, Germany

KH_2PO_4	Roth, Karlsruhe, Germany
[2]Components vitamin mix	
Choline chloride (50% S)	Bergophor, Kulmbach, Germany
Microvit A Promix 1000, 1.000.000 I.E./g	Bergophor, Kulmbach, Germany
Microvit B1 Promix Thiamine Mono, 100%	Bergophor, Kulmbach, Germany
Microvit B2 Supra 80, 80%	Bergophor, Kulmbach, Germany
Microvit B3 Promix Niacin 99.0%	Bergophor, Kulmbach, Germany
Microvit B5 Promix D-Calpan, 100%	Bergophor, Kulmbach, Germany
Microvit B6 Promix Pyridoxine, 98%	Bergophor, Kulmbach, Germany
Microvit H Promix Biotin, 2%	Bergophor, Kulmbach, Germany
Microvit B9 Supra 100, 100%	Bergophor, Kulmbach, Germany
Microvit B12 Promix 1000, 1.0%	Bergophor, Kulmbach, Germany
Microvit D3 Promix 500, 500.000 I.E./g	Bergophor, Kulmbach, Germany
Microvit E Promix 50, 500 I.E./g	Bergophor, Kulmbach, Germany
Microvit K3 Promix MNB 51%	Bergophor, Kulmbach, Germany

Table S2 Characteristics of gene specific primers used for qPCR

Gene symbol	Primer sequence (5´-3´, forward, reverse)	Product size (bp)	NCBI GenBank accession number
Reference genes			
Atp5b	GCCAGAGACTATGCGGCGCA, CCCCCAAATGCTGGGCCACC	187	NM_016774
Canx	GTCCCCGGGAGGCTCGAGATAGAT, ACCTCCCCTGTTGGAACTGGAGC	234	NM_007597
Cyc1	CGCAATGGAAGCTGCCGGGA, CGCAATGGAAGCTGCCGGGA	99	NM_025567
Target genes			
Acadl	CACAGGCCCTGGCTTCAGCC, ATGCCATGGGCAGGCGATCG	303	NM_007381
Acadm	AGGTTTCAAGATCGCAATGG, CTCCTTGGTGCTCCACTAGC	152	NM_007382
Atf4	TTCCTCGAATCCAGCAAAGCCC, CTCCAACATCCAATCTGTCCGG	286	NM_009716
Casp12	GATGGAGAAGGAGGGACGAACAC, CACCAGGAATGTGCTGTCTGAGG	235	NM_009808
Clpp	CACACCAAGCAGAGCCTACA, TCCAAGATGCCAAACTCTTG		NM_017393
Cox4i1	CTTCGAGCACATGGGAGTGT, ATCAGAACGAGCGCAGTGAA	296	NM_009941
Ddit3	AGCCAGAATAACAGCCGGAACC, GGGACTCAGCTGCCATGACTG	117	NM_007837
Dnajc3	CTGCATGGACACAGGTCCCAG, AGGCTGGACACCCCTACCTC	217	NM_008929
Gadd34	GCGGCTCAGATTGTTCAAAGCC, AGATGGGTTTCTAAGGCGTGCC	271	NM_008654
Gapdh	CAACGACCCCTTCATTGAC, AGACTCCACGACATACTCAG	196	NM_001289726
Gpx1	GTCCACCGTGTATGCCTTCT, TCTGCAGATCGTTCATCTCG	152	NM_008160
Gys1	AGGGGATGAATGGGGTGACA, CATTCGTGGAAGTGGGCAAC	384	NM_030678
Herpud1	CGCAGTTGGAGTGTGAGTCGC, ACCCTTTGTGCTGGTTTCTGGC	216	NM_022331
Hmox1	GATTTGTCTGAGGCCTTGAAG, CTTAAAGCCTTCTCTGGACAC	111	NM_010442

Hspa5	TCATCGGACGCACTTGGAATGAC, CGCTGGGCATCATTGAAGTAAGC	250	NM_001163434
Hsp10	CTGACAGGCTCAATCTCTCCAC, AGGTGGCATTATGCTTCCAG	108	NM_008303
Hsp90b1	CCTGCTGACCTTCGGGTTCG, CTGTCCTTGAGCCTTCTCGGC	98	NM_011631
Ldha	CTTGGCGCTCTACTTGCTGT, CTCATCCGCCAAGTCCTTCA	334	NM_001136069
Pdia4	CCGTTGACTATGATGGCTCCAGG, GGTCTGTCTGTTCGGTGGCG	285	NM_009787
Pfkm	CCAGTGCATCTTAACCGACCAT, TCGGGCACTTCCAATCACTG	309	NM_021514
Phka1	TGAGTGGAGACGGGGTCTTT, TCACGGTCAGCATTTTTGCG	404	NM_008832
Ppargc1a	GACCCTCCTCACACCAAAC, GCGACTGCGGTTGTGTATG	91	XM_006503779
Pygm	CTCCAGGACATCATCCGACG, TTCACTGTTACGTCCCAGGC	200	NM_011224
Slc25A20	TGGACACTGTTGCTGAGA, TTGGCCAAAGGTATCGAG	225	NM_020520
Sod1	GAGACCTGGGCAATGTGACT, GTTTACTGCGCAATCCCAAT	220	NM_011434
Txnrd1	GACTGGCAGCAGCTAAGGAGG, GCTTGTCCGAGCAAAGCTGC	166	NM_001042523
